# The return of the Traditional Birth Attendant

**DOI:** 10.7189/jogh.06.020302

**Published:** 2016-12

**Authors:** Karen Lane, Jayne Garrod

**Affiliations:** School of Humanities and Social Science, Faculty of Arts and Education, Deakin University, Burwood, Victoria, Australia

The World Health Organization has noted much progress towards the realisation of Millennium Development Goals related to maternal and child health. Eighty percent of women in many developing economies now receive at least one visit during pregnancy by a skilled birth attendant (although only 52% had the recommended four visits), and 68% of women across developing regions receive skilled health attendant care (up from 56% in 1990). However, disparities follow regional and urban–rural gaps. Sub–Saharan Africa and Southern Asia lag behind other regions in the provision of antenatal care and skilled attendance at birth (although typically attended by a family member or villager) and over 32 million of the 40 million births not attended by skilled health personnel in 2012 occurred in rural areas. Overall, one–quarter of women in developing nations still birth alone or with a relative to assist them.

While increased numbers of medically–trained midwives and health workers or midwife assistants would increase coverage by up to 40%, these are longer–term solutions. In the short term, gross disparities in services in some resource–poor areas have been alleviated by recruiting Traditional Birth Attendants (TBAs) re–trained in emergency obstetric skills to deal with emergency situations and to refer women onto health facilities when necessary. Samoa and Bangladesh are examples. For many women for a range of reasons TBAs are preferable to hospital care. It therefore makes sense to recognise their place within maternity care, to offer basic and ongoing training and to set up registration procedures thus better ensuring the monitoring of outcomes. Incorporating TBAs into the formal health care system would meet both physiological and relational components of birth. In terms of the latter, TBAs would act as cultural brokers between Western and traditional cosmologies and provide women with continuity of care from a known carer; in the West a demonstrably simple but effective intervention promoting physiological safety and reducing the need for higher level medical interventions.

## HUMAN RIGHTS, MILLENNIUM DEVELOPMENT GOALS AND MATERNITY CARE

Despite reiterations of human rights declarations and conventions from 1945 to the present, including revised Millennium Development Goals (MDG) calling for a 75% reductions in Maternal Mortality Rates (MMRs) by 2015, outcomes in some areas remain significantly unchanged leading to a cumulative downward cycle of poor health for offspring, sustained poverty and incremental social disadvantage. MDG3 aims to promote gender equality and empower women, MDG4 calls for reduction of infant mortality by two thirds; and MDG5 calls for improvement to maternal health. None has been realized to expected levels. Baseline data from 1990 figures showed that over a 15–year period to 2005 maternal deaths decreased by 5.4%; an average of 0.4% per annum although none of the eight regions targeted achieved the goal of 5.5% p.a. reduction Very little progress was made in sub–Saharan Africa and Southern Asia and although progress was made compared to baseline data the level of skilled birth attendants (SBAs) remained low. Other regions increased their provision of SBAs but failed to catch up to the developed world and fell well below the targeted 80% reduction in MMRs and IMRs. Only 47% of women received four antenatal visits during pregnancy – an unchanged percentage from baseline – although more women (79% up from 64%) received some antenatal care (MDG 5B). Taken as a whole, developing countries increased Skilled Birth Attendance (SBA) from 43% at baseline to 57% but the target of 90% by 2015 has remained an unrealised aspiration and will remain so for the near future [1,2].

**Figure:**
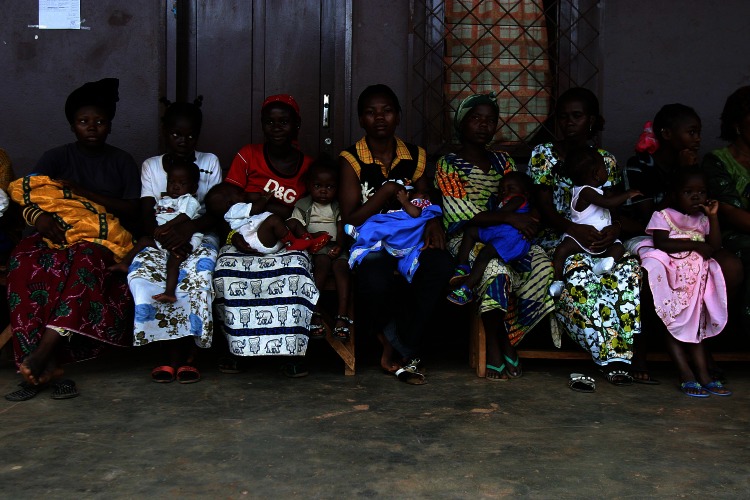
Photo: By hdptcar (Flickr User) [CC BY 2.0 (http://creativecommons.org/licenses/by/2.0)], via Wikimedia Commons

According to the World Health Organization (WHO), fresh remedial strategies and pragmatic advice is required around cost–effective interventions to address the major causes of child mortality–pneumonia, diarrhoea, birth asphyxia, preterm birth/low birth weight and neonatal infections–and maternal mortality–post–partum haemorrhage (PPH) hypertensive disease, obstructed labour and complications associated with unsafe abortion Although data are approximate only, sources indicate that around 99% of maternal deaths occur in developing countries (and the majority of those in Northern Africa and Southern Asia) [1,3]. There have been substantial advances in many countries during the past 40 years, viz. Thailand, Malaysia, Sri Lanka and Egypt and Honduras. In rural Bangladesh, where women continue to birth at home without a professional attendant, the MMR declined by 25% over 25 years from 1976 to 2001 due to better access to surgical obstetric care, reduced fertility, lower abortion rates and improved health generally [1]. Progress was also made in eastern and southern Africa where maternal mortality fell from 740 to 410 deaths per 100 000 births. However, advances are slow and most nations in the region failed to realise the MDG5 goal to reduce maternal deaths by three–quarters by 2015. Child mortality is closely related to maternal mortality but no change has occurred in the same region where over half of the births occur at home without skilled assistance or a postnatal visit within 48 hours after birth. Cord care, infection control, management of emergencies, especially postpartum haemorrhage–the leading cause of maternal death–and referral to expert care are obvious remedial measures not made readily available to these women and families.

## THEN WHY BIRTH AT HOME?

Around three–quarters (74%) of maternal deaths are considered to be preventable although women commonly face barriers in accessing facility–based birth. Shiferaw et al confirmed that 78% of lower–income and lower–educated women in Ethiopia chose a TBA; 42% reported it was not necessary to attend a facility (often meaning their husbands considered it unnecessary), 22% attributed the decision to the cost of formal maternity care, 36% said facility birth was not customary and 8% cited long distances or lack of transport [4]. Other women in Rosen et al’s direct observational study of over 2000 births in facilities across fifteen poor–resourced nations found much disrespect and abuse in facility–based care, mostly in the form of abandonment and neglect [5]. The same studies found that appropriately trained TBAs represent a viable solution to the new call by WHO for innovative and pragmatic arrangements to provide all women with a skilled birth attendant.

## REMEDIAL STRATEGIES TO REDUCE MATERNAL MORTALITY RATES AND INFANT MORTALITY RATES

In this regard, health visitor training and training of new entrants to Bachelor of Midwifery programs comprise longer–term interventions given the shortfall in the numbers of females at primary, secondary and tertiary level education especially in sub–Saharan Africa, Oceania, Western Asia and Northern Africa (although girls enrolments have increased in Latin America and the Caribbean). More immediate measures include family spacing, contraceptive use and the provision of at least four antenatal visits. UNFPA [3] recommended recruitment of village midwives: a surprising initiative given the joint WHO, UNFPA and Safe Motherhood (Safe Motherhood Initiative (SMI) comprising United Nations, ARROW and the White Ribbon Alliance) statement in the 1990s recommending scaling down Traditional Birth Attendants (TBAs) on the grounds they failed to reduce MMRs and IMRs over the accounting period.

However, as others have noted since, the WHO/UNFPA/SMI decision to deter TBA integration into the formal maternity care system in the early 1990s was hastily conceived [6]. For many years in the 1970s and 80s, TBAs were germane to equity considerations. WHO–sponsored programs had trained them in antenatal, intrapartum and postpartum skills to detect early complications, ensure timely referrals and to reduce infection and postpartum haemorrhage (PPH). By the 1990s, however, WHO declared the TBA initiative had failed and henceforth only medically–trained midwives or nurse practitioners would be installed because only they could identify obstetric risks and be trusted to refer women onto a clinic or hospital [7].

The problem with rescinding TBA endorsement was that WHO had failed to establish a baseline measurement in the 1970s which meant it was impossible to fairly judge TBA performance after a twenty year trial. And accurate recording of birth data remains a significant problem in assessing efficacy of any initiative [8]. The complexity of the problem grows when factoring in the social determinants of health such as poverty, illiteracy, lack of easy access to facilities including impassable roads, lack of transport, poor general health of mothers and the involvement of other community actors, such as family and village elders as causal agents. In summary, developing countries have diverse and complex historical, social, cultural and geographical barriers that need to be inserted into the causal matrix of risk factors accounting for MMRs and IMRs [9]. It also means there is no one strategy that will alleviate the problem although the medical solution generally is a rapid shift towards professionalization of care–givers in the antenatal, intrapartum and postnatal periods [10].

## WHY TBAs?

From the medical perspective, tackling maternal mortality requires upscaling the availability of medically–trained teams of midwives and midwife assistants in health facilities attending mainly to intrapartum care but also to antenatal and postpartum care, family planning and safe abortion [11]. Measures to manage PPH are tantamount since it is the single most important risk factor in determining high or low MMRs. But the problem remains in filling the gaps in the interim period between now and accreditation especially when the pool of educated school leavers remains small.

Some nations such as Pakistan, Bangladesh and Samoa not only educate TBAs but are prepared to recognise their tacit skills and knowledge including the use of traditional bush remedies. Further, their embeddedness in traditional cosmologies, cultural rites and social protocols means they act as cultural brokers for women who typically blend medical care with traditional TBA care [12,13] for a range of reasons–social, cultural, financial, and medical.

Some medical commentators are reluctant to endorse the use of other than strictly medically–trained professionals on the grounds they lack academic training, clinical opportunities and a supportive medical environment. However, as UNFPA later realised, TBAs could provide the pragmatic response urgently sought by WHO to meet 2015 Millennial Development Goals and Beyond if trained in the use of medications such as Misoprostol for abortion, medical management of miscarriage, induction of labour, cervical ripening before surgical procedures, and the treatment of postpartum haemorrhage. TBAs could also be trained to use the non–pneumatic AntiShock Garment for postpartum haemorrhage and the haemoglobin colour scale for screening anaemia in pregnancy [14]. Other preventive medications include low dose aspirin and calcium supplementation to reduce the risk of pre–eclampsia. With the addition of other key medical supplies such as antibiotics, magnesium sulphate for eclampsia and safe blood supplies, TBAs could provide greater coverage in areas of greatest deprivation [15,16].

Whilst they could not replicate high level obstetric techniques such as caesarean section or administer an oxytocin drip to arrest PPH or deal with drugs that require refrigeration, other medications available in tablet form, like Misoprostol, could be administered with good effect. The argument is that better coverage in poor resource nations could be achieved by recognising the skills of those already on the ground who, for many women, are currently the carer of choice for a variety of reasons. There is also the point that regardless of whether they are endorsed officially by the health system, or not, women continue to consult them for birth and physical ailments because they often double as village healers using traditional medicines harvested from the bush. In remote areas of Vanuatu, for example, while not endorsed by the health system, rural women typically split their care between the local TBA and the hospital/health post and many of the midwives and doctors recognised their skills and expertise [13]. In Samoa, TBAs are recognised as valuable adjuncts to the formal maternity care system [12] working collaboratively with staff at Level 1 EmOC facilities (health centres with capacities for administering oxytocin, antibiotics, anticonvulsants, manual removal of the placenta, vaginal delivery and resuscitation) and at Level 2 EmOC facilities (that carry out caesarean section and safe blood transfusion) [17].

## THE RELATIONAL COMPONENTS OF BIRTH

But there is another issue. Whilst the WHO and UNFPA provision of skilled medical care could decrease 79.9% of maternal deaths, medical expertise comprises only one aspect of “a good birth” [3,18]. Quality of care extends to the *relational* components of birth, namely a known and trusted carer (a rare event still in most Western arrangements), respectful dialogue and practices, consensual care, tolerance for informed *dissent* and provision of information and privacy but these aspects of birth have received less attention from policy–makers and providers [19] obviously because of the urgent need to address the physiological aspects of ensuring safe birth. In the case of rural and remote mothers who are either reluctant or unable to attend a health facility (see above), TBAs are ideally placed to act as cultural brokers between Western medical and local, situated knowledges. The utility of relational components (considerations of individual preferences, sacred practices prioritised by the local community and shared cultural values) is affirmed by the success produced by midwifery–led models offering one–to–one care with a known carer [20].

The Department of Health in UK is now committed to providing universal one to one care in labour to promote normality and reduction in intervention such as caesarean section as are Scottish and Welsh governments who found that women preferred continuous one–to–one care from a known midwife they trusted (not just a midwife). In Australia, women assigned to caseload models (continuous care in all phases from a known and trusted carer, with some backup from others) achieved higher rates of spontaneous vaginal birth, lower CS, epidural, episiotomy rates and babies were less like to be admitted to special care or neonatal intensive unit. Cochrane Collaboration reviews have concluded one–to–one care resulted in spontaneous vaginal birth, shorter labour, less use of analgesia in labour and babies with a low Apgar score at 5 minutes and generally reduced intervention [20]. Although the studies cited were conducted in Western locations, the evidence is convincing that a known carer especially one trained in emergency medical and obstetric techniques not only expedites safe birth but also fulfils human rights criteria, namely choice and interpersonal respect while facilitating ease of birth.

This is because bodies are not just physical entities but cultural resources that bend and move in relation to the social and cultural environment. Birth is an embodied phenomenon rather than a set of isolated physiological processes [15]. Respectful and collaborative practices are not simply “soft” add–ons but translate into better physiological outcomes. The UNFPA revised decision (based upon the Somalian review of maternity care and other more recent studies by Sibley et al [16]) found that TBAs were a valuable addition to the maternity care arsenal in developing economies because they enacted a horizontal model of knowledge where no one individual holds a dominant authoritative position and where the body is seen at the intersection of nature and culture.

In summary, given the need for immediate and pragmatic solutions to enduring problems facing remote and resource–poor women, TBAs are ideally placed geographically and culturally to administer basic but potentially life–saving medical and emergency care, to provide antenatal and postpartum care and to refer women with risk factors to formal health facilities. They could be registered and offered regular refresher courses thereby contributing to lower maternal and infant mortality and morbidity rates.
